# Cementless Mobile-Bearing Total Knee Arthroplasty: 10 Years Follow-Up

**DOI:** 10.7759/cureus.38259

**Published:** 2023-04-28

**Authors:** Ahmad G Abdallatif, Corrina Winkworth, Nadim Aslam

**Affiliations:** 1 Trauma and Orthopaedics, Worcester Royal Hospital, Worcester, GBR; 2 Trauma and Orthopaedics, Worcester Royal Hospital, Worcestershire, GBR

**Keywords:** two different age groups, joint prosthesis survivorship, oxford knee score, 10 years follow up, cementless total knee arthroplasty

## Abstract

Background

Although most TKR surgeries are cemented, the interest in cementless TKR has increased dramatically during the last few years because of the new generation of cementless prostheses and the increased number of young patients who need TKR.

Methods

Ten years of retrospective reviews of 80 patients who had cementless, complete rotating platform TKR (DePuy Synthes, Warsaw, Indiana) were performed. Patients were divided into two groups according to their age (above and below 70 years old). Functional outcomes at the final follow-up were evaluated clinically by filling out a satisfaction form, and the Oxford Knee Score as well as all medical and surgical complications were recorded for each patient.

Results

The 10-year cumulative implant survival rate was 100%, i.e. no patients had revision surgeries with no significant statistical difference between the two age groups. The 10-year evaluation rate was 90%.

Conclusion

The use of cementless TKA exhibited good survivability, long-term clinical and functional results, and no implant revision in various age groups, as well as a high satisfaction rate. There was no statistically significant difference between the results of different age groups.

## Introduction

Knee osteoarthritis (OA) affects 3.48% of the global population, thus increasing the need for advanced total knee replacement (TKR) [1]. TKR has become one of the most popular orthopedic surgeries for the management of knee osteoarthritis, with 272,133 cases reported in the UK between 2018 and 2020. The total knee arthroplasty (TKA) procedure is associated with a high satisfaction rate and low morbidity and mortality rates.

Cemented TKR is the most widespread method of TKR fixation, although the interest in cementless TKR has increased recently because of the new generation of cementless prostheses. The first generation of cementless TKA demonstrates early loosening related to micromotion in short- and mid-term studies. In contrast, the newer generation of implants is ideally bone-preserving and provides the necessary mechanical stability to obtain rapid and long-lasting osseointegration. The new generations have good geometry, an effective osteoconductive surface (porous coating, hydroxyapatite-coated, plasma spray), and adequate early stable fixation properties. The high fixation strength of the newer cementless implants prevents the need for supplemental screw fixation and the potential for screw track osteolysis observed in previous designs [2-6].

With the new generation of cementless implants and the increased number of TKR surgeries carried out on young patients (below 70 years of age) with longer life expectancy and higher postoperative activities, a significantly increased interest was observed in using the cementless technique as an alternative to the cemented technique that has a high failure rate at National Joint Registry (NJR) with young patients compared with old ones [7].

The theoretical benefits of the cementless technique include shorter operating time, the preservation of bone stock, which is important in revisions, and the prevention of complications associated with bone cement use [8].

Cementless implants are more expensive than cemented ones because of the high technology required to produce bioactive surfaces. Proponents of the cemented technique maintain that it is not reasonable to use expensive implants that give the same outcome as cheaper ones. However, using cementless implants saves time, includes tourniquet pneumatic ischemic time with less bone resection, and makes revision easier in failure cases. In some studies comparing the cost of the two different techniques, no significant difference in cost was observed between using the cemented and cementless implants [9,10].

One of the main concerns about using the cementless technique is regarding the ability of osteoporotic bone to provide adequate ingrowth and resist aseptic loosening. P. Dixon et al. compared 135 patients above 75 years of age and treated with cementless TKR with 423 patients below 75 years of age and treated with cementless TKR. They concluded that elderly patients do just as well as the younger group using this hydroxyapatite-coated, cementless total knee replacement after five years of follow-up [11].

Many studies, including level-one studies, have been undertaken to compare the two techniques [12-16]. The systematic review and meta-analyses of these studies indicate no significant difference in postoperative function or revision rate between cemented and cementless TKR implants up to 16.6 years of follow-up (mean 8.4 years) [17].

The present study focused on the implant survival rate and clinical outcome after 10 years of follow-up, comparing two different age groups: those above 70 years of age and those below. The endpoint defined as revision for any further operative intervention included a change of the polyethylene insert. Secondary objectives were to quantify functional outcomes at 10 years (Oxford Knee Score) as well as evaluation rate, intraoperative complications, and mortality.

## Materials and methods

**Figure 1:**
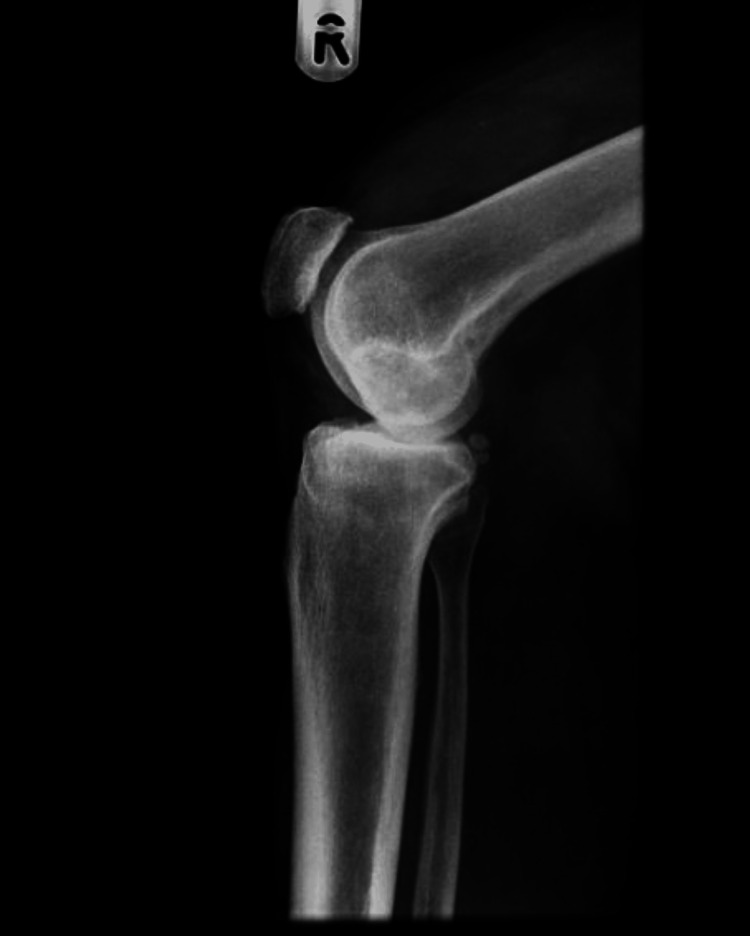
Preoperative X-ray, lateral view

**Figure 2:**
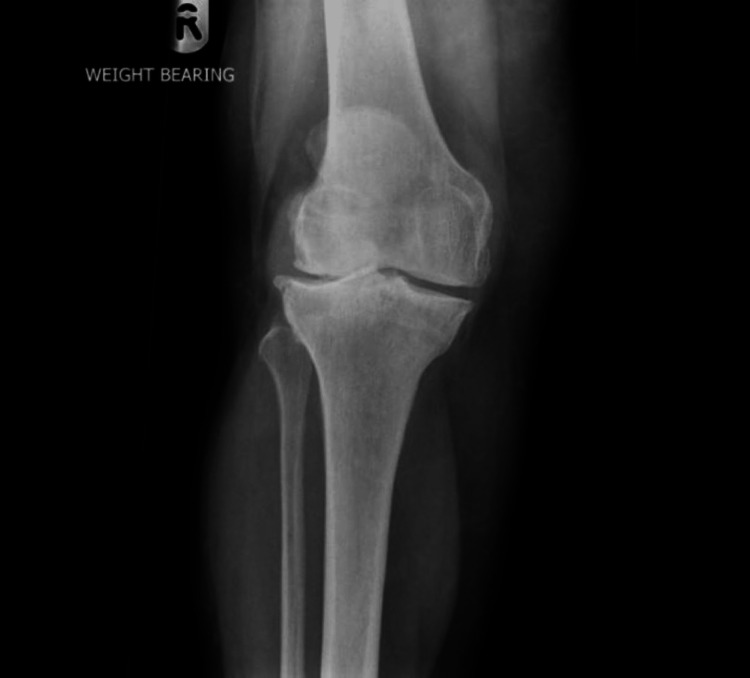
Preoperative X-ray, AP view AP: anteroposterior

A total of 97 patients underwent cementless total knee replacement between January 2010 and January 2011. Three patients could not be reached, and 14 patients died within 10 years of surgery. All patients received the same prosthesis - DePuy Synthes LCS complete (DePuy Synthes, Warsaw, Indiana), and their surgery was performed by a single senior surgeon. Patients had a preoperative X-ray (Figures 1, 2).

All surgical procedures involved a midline skin incision and then a medial parapatellar approach for the knee joint. The flexion and extension gaps were balanced by employing the gap-balancing technique. All patients had surgery using intramedullary femoral and tibial cutting guides. The range of motion, stability, and patellar tracking were assessed before and after implantation. No surgery involved any patellar resurfacing.

**Figure 3:**
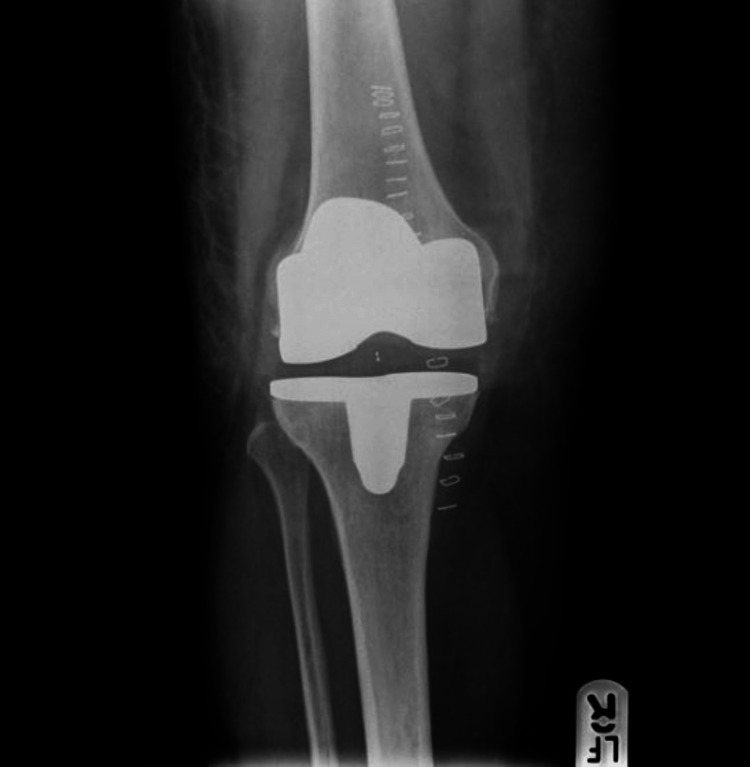
Postoperative X-ray, AP view AP: anteroposterior

**Figure 4:**
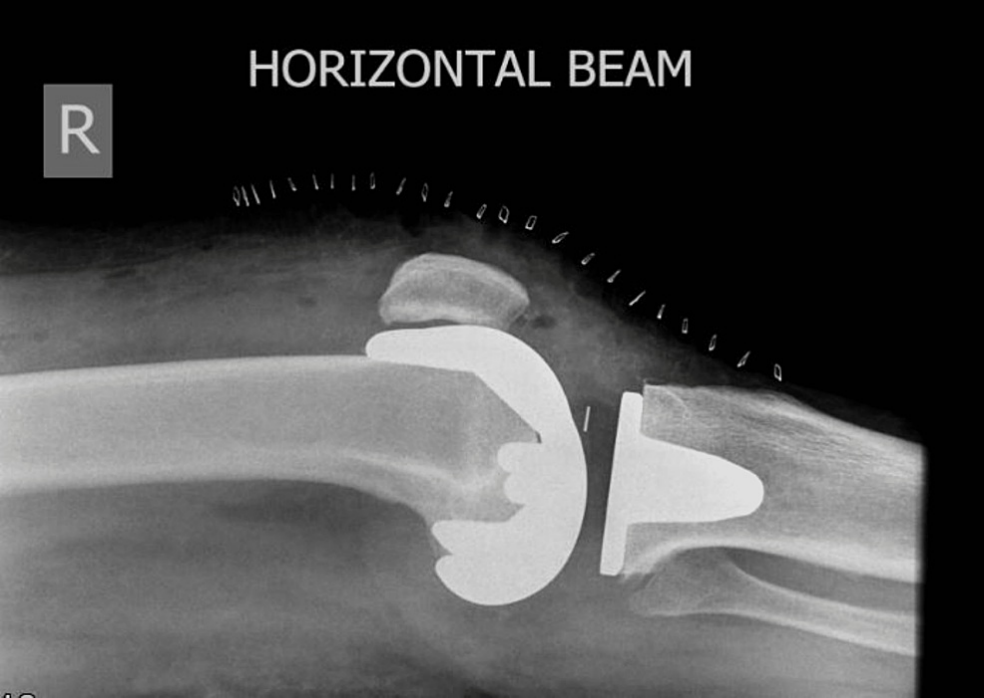
Postoperative X-ray, lateral view

**Figure 5:**
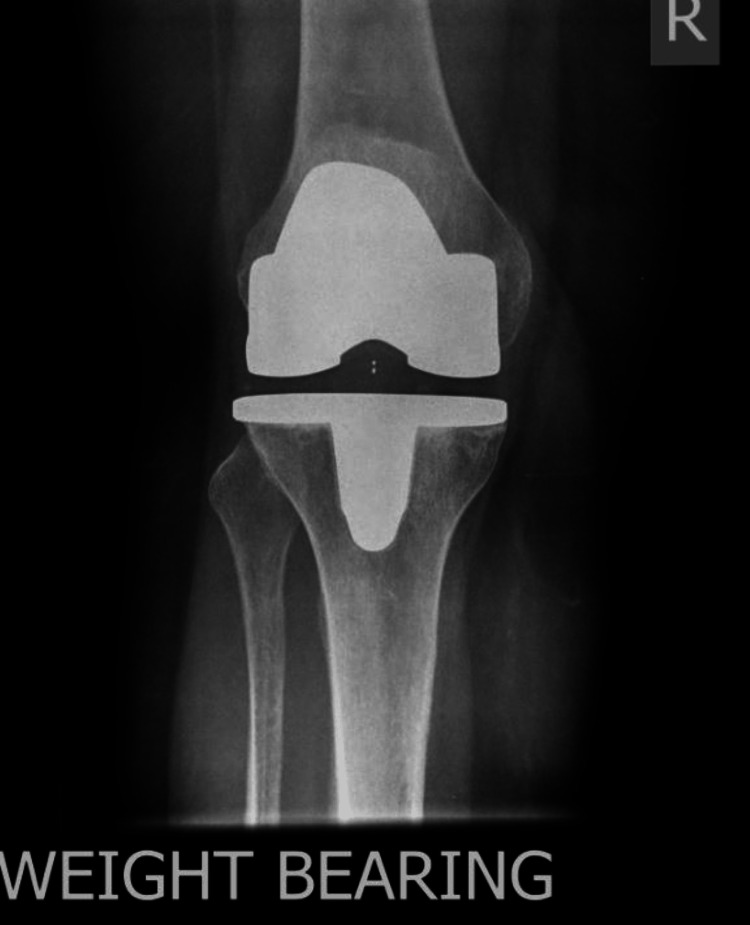
One year postoperative X-ray, AP view AP: anteroposterior

**Figure 6:**
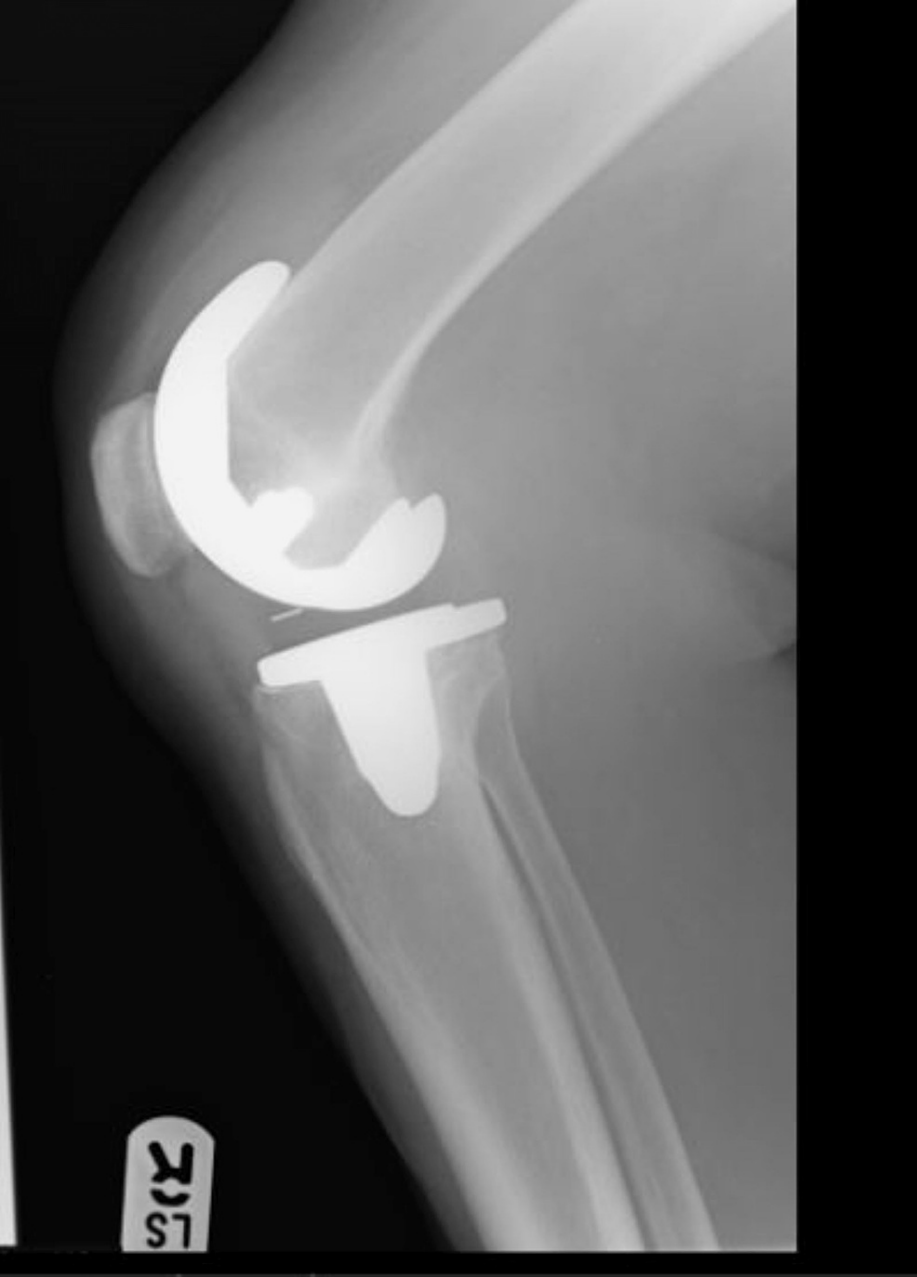
One year postoperative X-ray, lateral view

A standard postoperative X-ray (Figures 3, 4) was obtained, and a physical therapy exercise program with full weight-bearing and range-of-motion exercises was started a day after surgery and prior to hospital discharge. An X-ray was requested at the last follow-up appointment (Figures 5, 6). The same surgical team monitored every patient who survived after surgery. The minimum follow-up period for patients who were still alive was 10 years.

The date of procedure, most recent check-up, and the case-specific analysis of failures resulting in revision comprised the data fields for implant survival data. Failure was defined as the need to change components (including spacers) or undergo a revision procedure. If the follow-up was shorter than 10 years, patients were telephonically invited to visit the clinic or submit postal questionnaires. Revision data were verified by the National Joint Registry to detect revisions performed at other centers.

For secondary outcomes, the case note review included clinic letters that collated data on complications, the Oxford knee score (as patient-reported outcome measures), X-ray radiographs, and additional treatment. Mortality data were obtained from the hospital mortality register.

All patients provided either verbal or written authorization for using the data gathered during this study. The patients were classified into two primary groups: those above 70 years of age and those below.

Statistical analysis

An implant revision was the endpoint. The paired student’s t-test was used to compare normally distributed data. The Mann-Whitney U test was used for non-parametric distributions, and medians were compared using U tests. Further, 95% confidence intervals (CIs) were employed, and the level of significance for each analysis was set at p = 0.05.

## Results

Overall, 97 surgeries were performed with cementless DePuy Synthes LCS. The mean age of the patients was 72 years (65-88). American Society of Anesthesiologists (ASA) was used as a surrogate for comorbidities: ASA 1 - 36%, ASA 2 - 63%, and ASA 3 - 1%. At 10 years after surgery, three patients were lost to follow-up and 14 patients had died, none of them in the first two years postoperative time. The study included 80 patients: 35 males and 45 females. Forty-three patients were under the age of 70, and 37 were above, with 72 being the average age.

Survivorship

**Figure 7:**
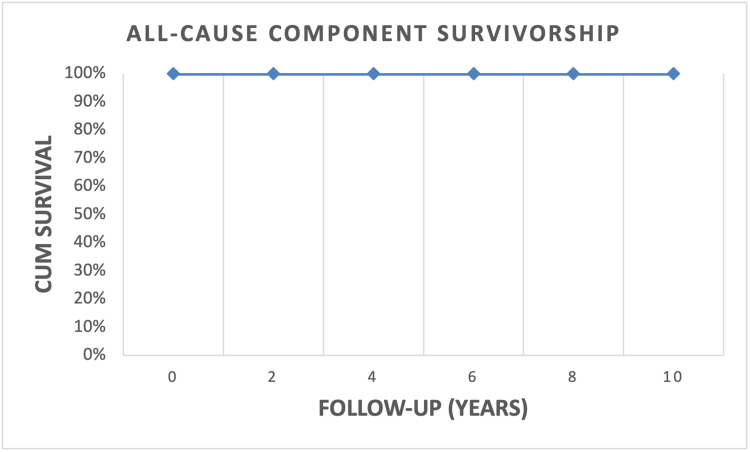
Kaplan-Meier survivorship

The cohort of patients had an aseptic survivorship of 100% (Figure 7). No aseptic failures were noted, and no revision surgeries were performed as per the latest follow-up visit. As none of the patients in this group had deep periprosthetic infections, the overall survivorship rate was 100%.

Revision rates

No patient underwent revision surgery within 10 years of index surgery.

Functional outcomes

The average postoperative Oxford score was 47.9125, with no statistically significant difference between the two age groups (p < 0.001) (Table 1).

**Table 1:** Postoperative Oxford Knee Scores and the average score recorded for patients (number and percentage) with cementless TKR.

Postoperative Oxford Knee Score	Score	Number	Percentage (%)
	44	1	1.25
	46	1	1.25
	47	1	1.25
	48	77	96.25
Average score	47.9125		

At 10 years of follow-up, the average Oxford Knee Score was 40, compared with the postoperative score of 47. The average Oxford score for the group above 70 years of age at the 10 years follow-up was 39.8 as compared with 40.3 for the younger group, with no significant statistical difference (p < 0.05) (Table 2).

**Table 2:** Ten-year postoperative Oxford Knee Scores

	Age < 70		Age > 70		Total	
Score	Number	Percentage (% out of 43)	Number	Percentage (% out of 37)	Number	Percentage (% out of 80)
48	2	4.65	5	13.51	7	8.75
47	2	4.65	4	10.81	6	7.5
46	4	9.30	1	2.70	5	6.25
45	2	4.65	2	5.40	4	5
44	2	4.65	1	2.70	3	3.75
43	3	6.97	4	10.81	7	8.75
42	8	18.60	5	13.51	13	16.25
41	4	9.30	1	2.70	5	6.25
40	3	6.97	5	13.51	8	10
39	2	4.65	1	2.70	3	3.75
38	3	6.97	1	2.70	4	5
34	1	2.32			1	1.25
33	1	2.32	1	2.70	2	2.5
32			1	2.70	1	1.25
31	1	2.32	-	-	1	1.25
30	1	2.32	-	-	1	1.25
28	4	9.30	-	-	4	5
26	-	-	1	2.70	1	1.25
18	-	-	1	2.70	1	1.25
15	-	-	1	2.70	1	1.25
10	-	-	2	5.40	2	2.5
Average score	40.3023		39.2432		39.8125	

All patients were asked to provide an overall evaluation of their experience with the surgery: 45 (56.25%) patients, including 23 from the older group, described their experience as excellent, 17 (21.25%), including seven from the older group, described it as very good, 10 (12.5%) patients, including four from the older group, described it as good, and eight (10%) patients, which included three from the older group, described it as fair (Table 3).

**Table 3:** Patient evaluation of the cementless TKR procedure after 10 years TKR: total knee replacement

Patient evaluation after 10 years	Grade	Number of patients	Percentage (%)
	Excellent	45	56.25
	Very good	17	21.25
	Good	10	12.5
	Fair	8	10

Mortality

Fourteen patients had died within 10 years of the surgery. Their cause of death was not investigated further; however, it was determined that none of the deaths were related to immediate surgical causes. None were in the first two years postoperative.

## Discussion

TKA with cementless fixation was developed to diminish cement-related difficulties, potentially maintain the local bone stock, and lengthen implant survival. Although the initial designs were associated with early failures, technological advancements have produced new implants and biomaterials that hasten implant osseointegration, which could result in an increase in the long-term survivability of cementless implants. Moreover, because of the increasing number of younger, more active patients requiring TKA, cementless fixation may be the best and most appropriate modality that decreases the risk of revision surgery. According to the findings of the current study, patients younger than 70 years who received cementless TKA had a 100% implant survival rate as well as outstanding functional outcome scores and range of motion at a mean four-year follow-up [13-17].

The long-term success of both cementless and cemented TKR depends on following the guidelines on managing soft-tissue alignments and bony cuts. In the current study, procedures depended on femoral and tibial intramedullary alignment for femoral and tibial bony cuts [18].

For both cemented and uncemented TKR, aseptic loosening of the tibia is the main concern. This concern is greater in the old population. Ten-year survivorship in this series is better compared with the best-reported cemented series [19-21].

Numerous studies have demonstrated excellent outcomes in patients after cementless TKA using newer-generation implants. Lizaur-Utrilla et al. performed 45 cementless and 48 cemented TKAs in patients who were younger than 55 years and had a mean follow-up of seven years [22]. They reported that the nine-year survivorship for aseptic failure was 94% in the cementless group and 90% in the cemented group. Moreover, by the latest follow-up, the cementless TKA group had significantly better knees with better Knee Society function scores (94 vs. 89 points, P = 0.022) and pain scores (47 vs. 44 points, P = 0.024) compared with the cemented TKA group. Jared et al. examined a total of 134 patients (142 TKAs) older than 75 years at a single institution with a mean follow-up of four years [23]. The aseptic implant survivorship was 99.3% (95% CI: 7.9-8.1), and the all-cause implant survivorship was 98.6% (95% CI: 7.9-8.1). There was one aseptic revision and one septic revision, and at the latest follow-up, the mean Knee Society pain score was 93 points (range: 80-100 points), and the mean Knee Society function score was 84 points (range: 70-90 points).

This paper encompasses two controversial issues within TKA: the efficacy of cementless TKR in providing a good outcome in comparison with cemented TKA in the long term and the comparison of the results between the two different age groups (above and below 70 years of age).

The current study had some limitations. The evaluation only included a cohort of patients who received cementless TKA and did not compare the outcomes to those of the cemented option. Moreover, this study was performed at a single institution. The strong points of this study are the large sample size and the long-term follow-up.

To evaluate the outcome of the cementless technique after 10 years of follow-up, two main parameters were employed: the Oxford Knee Score and the overall patient satisfaction and evaluation. Since the Oxford Hip and Knee Scores have been evaluated independently and found to be the best and most reliable systems for the assessment of hip and knee replacement, respectively, using them in this study was deemed advantageous [24-27].

The study also depended on the detection of patient satisfaction after 10 years of the surgery. Ware et al. were the first to describe the concept of patient satisfaction in 1873. Patient satisfaction after TKR can be associated with patients’ expectations, pain management, and functional improvement [28].

Baker et al. reviewed the data from the National Joint Registry for England and Wales; these data show that 71% of the patients had improvement in knee symptoms, but only 22% rated the outcome as “excellent” while 29 were not satisfied with the results [29].

## Conclusions

The use of cementless TKA exhibited good survivability, long-term clinical and functional results, and no implant revision in various age groups, as well as a high satisfaction rate. There was no statistically significant difference between the results of different age groups. Hence, cementless TKA may be a viable alternative for individuals of any age that does not lead to any significant complication. Further study, including a control randomized multi-center study, is still needed to support the obtained results and shed further light upon the differences between the implementation of both cementless and cemented techniques. A longer follow-up study (15 years) for cementless total knee replacement will also be needed for a better evaluation of this technique.
